# Supplementary material for: Microglia Are Necessary to Regulate Sleep after an Immune Challenge
Source: Biology (Basel). 2022 Aug 19;11(8):1241. doi: 10.3390/biology11081241 (PMC9405260; doi:10.3390/biology11081241)
Supplement: Supplementary file 1 [file biology-11-01241-s001.zip › biology-1779932-supplementary.pdf]

**Supplementary Figure**

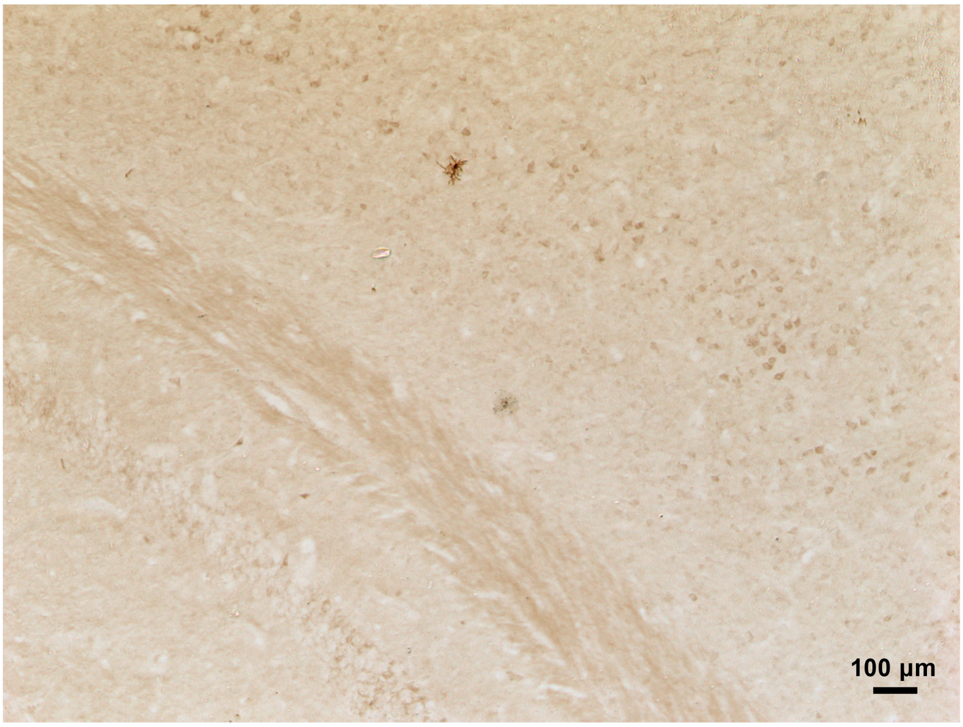


**Figure S1:** Microglia were depleted after a 21-day administration of PLX. Photomicrograph of Iba-1-stained microglia in the cortex. Scale bar = 100 µm.

## Supplementary Tables

**Table S1:** Differences between estimated marginal means (Δ), corresponding *p*-values, and effect sizes (*d*) from generalized linear mixed models for percent sleep, mean bout length, and cumulative sleep during weeks 1, 2, and 3 of the microglia depletion period. Estimated nonparametric Spearman's rank correlation (ρ) and corresponding *p*-values for temporal pattern differences in percent sleep and mean bout length. * indicates statistically significant differences in means.

**Light Period**

| | **Mean Δ** | **Δ *p*-value** | **Δ *d*** | **ρ** | **ρ *p*-value** |
|---|---|---|---|---|---|
| *Percent Sleep* | | | | | |
| Week 1 | 5.1% | **0.02*** | 0.10 | 0.91 | <0.0001 |
| Week 2 | 2.8% | 0.45 | 0.04 | 0.89 | <0.0001 |
| Week 3 | 2.2% | 0.62 | 0.05 | 0.82 | 0.0006 |
| *Mean Bout Length* | | | | | |
| Week 1 | 8.2 sec | **0.01*** | 0.02 | 0.85 | <0.0001 |
| Week 2 | 6.9 sec | 0.18 | 0.02 | 0.99 | <0.0001 |
| Week 3 | 8.1 sec | 0.15 | 0.03 | 0.78 | 0.001 |
| *Cumulative Sleep* | | | | | |
| Week 1 | 301 min | **0.02*** | 0.01 | – | – |
| Week 2 | 159 min | 0.53 | 0.006 | – | – |
| Week 3 | 130 min | 0.69 | 0.005 | – | – |

**Dark Period**

| | **Mean Δ** | **Δ *p*-value** | **Δ *d*** | **ρ** | **ρ *p*-value** |
|---|---|---|---|---|---|
| *Percent Sleep* | | | | | |
| Week 1 | 2.7% | 0.60 | 0.05 | 0.96 | <0.0001 |
| Week 2 | 2.6% | 0.61 | 0.03 | 0.98 | <0.0001 |
| Week 3 | 1.2% | 0.83 | 0.10 | 0.95 | <0.0001 |
| *Mean Bout Length* | | | | | |
| Week 1 | 3.2 sec | 0.63 | 0.06 | 0.90 | 0.0004 |
| Week 2 | 5.4 sec | 0.42 | 0.06 | 0.94 | <0.0001 |
| Week 3 | 8.0 sec | 0.29 | 0.04 | 0.92 | 0.0002 |
| *Cumulative Sleep* | | | | | |
| Week 1 | 143 min | 0.40 | 0.001 | – | – |
| Week 2 | 186 min | 0.15 | 0.002 | – | – |
| Week 3 | 193 min | 0.20 | 0.002 | – | – |

**Table S2:** Differences between estimated marginal means (Δ), corresponding *p*-values, and effect sizes (*d*) from generalized linear mixed models for percent sleep, mean bout length, and cumulative sleep during days 1, 2, 3, and 4 following the administration of lipopolysaccharide (LPS1). Estimated nonparametric Spearman's rank correlation (ρ) and corresponding *p*-values for temporal pattern differences in percent sleep and mean bout length. * indicates statistically significant differences in means; # indicates statistically significant temporal pattern differences.

**Light Period**

| | **Mean Δ** | **Δ *p*-value** | **Δ *d*** | **ρ** | **ρ *p*-value** |
|---|---|---|---|---|---|
| *Percent Sleep* | | | | | |
| Day 1 | 0.2% | 0.96 | 0.05 | **0.44#** | **0.12#** |
| Day 2 | 6.0% | 0.13 | 0.05 | 0.78 | 0.001 |
| Day 3 | 2.3% | 0.53 | 0.02 | **0.13#** | **0.65#** |
| Day 4 | 2.1% | 0.56 | 0.04 | 0.68 | 0.01 |
| *Mean Bout Length* | | | | | |
| Day 1 | 1.0 sec | 0.60 | 0.02 | -0.70 | 0.007 |
| Day 2 | 2.0 sec | 0.39 | 0.02 | 0.64 | 0.02 |
| Day 3 | 8.4 sec | **0.02*** | 0.02 | **0.11#** | **0.70#** |
| Day 4 | 1.4 sec | 0.78 | 0.01 | **0.51#** | **0.07#** |
| *Cumulative Sleep* | | | | | |
| Day 1 | 3 min | 0.99 | 0.0008 | – | – |
| Day 2 | 55 min | 0.22 | 0.001 | – | – |
| Day 3 | 10 min | 0.97 | 0.0002 | | |
| Day 4 | 10 min | 0.99 | 0.0002 | – | – |

**Dark Period**

| | **Mean Δ** | **Δ *p*-value** | **Δ *d*** | **ρ** | **ρ *p*-value** |
|---|---|---|---|---|---|
| *Percent Sleep* | | | | | |
| Day 1 | 15.1% | **0.0007*** | 0.05 | **0.58#** | **0.06#** |
| Day 2 | 11.1% | **0.02*** | 0.06 | 0.85 | 0.004 |
| Day 3 | 13.7% | 0.10 | 0.08 | 0.96 | <0.0001 |
| Day 4 | 10.3% | 0.11 | 0.04 | 0.92 | 0.0005 |
| *Mean Bout Length* | | | | | |
| Day 1 | 11.5 sec | **0.001*** | 0.02 | **0.60#** | **0.07#** |
| Day 2 | 1.0 sec | 0.85 | 0.03 | 0.65 | 0.04 |
| Day 3 | 9.3 sec | 0.18 | 0.01 | 0.93 | 0.0001 |
| Day 4 | 13.2 sec | **0.01*** | 0.01 | 0.84 | 0.004 |
| *Cumulative Sleep* | | | | | |
| Day 1 | 95 min | **0.0004*** | 0.03 | – | – |
| Day 2 | 65 min | **0.007*** | 0.02 | – | – |
| Day 3 | 91 min | **<0.0001*** | 0.04 | | |
| Day 4 | 79 min | **<0.0001*** | 0.05 | – | – |

**Table S3:** Differences between estimated marginal means (Δ), corresponding *p*-values, and effect sizes (*d*) from generalized linear mixed models for percent sleep, mean bout length, and cumulative sleep during weeks 1 and 2 of the microglia repopulation period. Week 2 comprised 3 days. Estimated nonparametric Spearman's rank correlation (ρ) and corresponding *p*-values for temporal pattern differences in percent sleep and mean bout length. * indicates statistically significant differences in means.

**Light Period**

| | **Mean Δ** | **Δ *p*-value** | **Δ *d*** | **ρ** | **ρ *p*-value** |
|---|---|---|---|---|---|
| *Percent Sleep* | | | | | |
| Week 1 | 2.6% | 0.56 | 0.03 | 0.75 | 0.003 |
| Week 2 | 2.4% | 0.63 | 0.02 | 0.81 | 0.0008 |
| *Mean Bout Length* | | | | | |
| Week 1 | 14.8 sec | **0.009*** | 0.03 | 0.98 | <0.0001 |
| Week 2 | 16.7 sec | 0.06 | 0.03 | 0.84 | 0.0002 |
| *Cumulative Sleep* | | | | | |
| Week 1 | 143 min | 0.94 | 0.002 | – | – |
| Week 2 | 43 min | 0.95 | 0.001 | – | – |

**Dark Period**

| | **Mean Δ** | **Δ *p*-value** | **Δ *d*** | **ρ** | **ρ *p*-value** |
|---|---|---|---|---|---|
| *Percent Sleep* | | | | | |
| Week 1 | 0.0% | 0.99 | 0.04 | 0.99 | <0.0001 |
| Week 2 | 1.7% | 0.74 | 0.11 | 0.94 | <0.0001 |
| *Mean Bout Length* | | | | | |
| Week 1 | 5.8 sec | 0.35 | 0.06 | 0.92 | 0.0005 |
| Week 2 | 4.2 sec | 0.55 | 0.07 | 0.90 | 0.0009 |
| *Cumulative Sleep* | | | | | |
| Week 1 | 128 min | 0.58 | 0.003 | – | – |
| Week 2 | 20 min | 0.88 | 0.01 | – | – |

**Table S4:** Differences between estimated marginal means (Δ), corresponding *p*-values, and effect sizes (*d*) from generalized linear mixed models for percent sleep, mean bout length, and cumulative sleep during days 1–7 following a second administration of lipopolysaccharide (LPS2). Estimated nonparametric Spearman's rank correlation (ρ) and corresponding *p*-values for temporal pattern differences in percent sleep and mean bout length. * indicates statistically significant differences in means; # indicates statistically significant temporal pattern differences.

**Light Period**

| | **Mean Δ** | **Δ *p*-value** | **Δ *d*** | **ρ** | **ρ *p*-value** |
|---|---|---|---|---|---|
| *Percent Sleep* | | | | | |
| Day 1 | 4.4% | 0.51 | 0.02 | 0.99 | <0.0001 |
| Day 2 | 5.0% | 0.42 | 0.03 | **0.54**[#] | **0.06**[#] |
| Day 3 | 3.7% | 0.41 | 0.04 | 0.89 | <0.0001 |
| Day 4 | 3.5% | 0.46 | 0.01 | **0.52**[#] | **0.06**[#] |
| Day 5 | 4.3% | 0.39 | 0.04 | 0.92 | <0.0001 |
| Day 6 | 3.8% | 0.46 | 0.03 | 0.94 | <0.0001 |
| Day 7 | 4.4% | 0.43 | 0.03 | 0.71 | 0.005 |
| *Mean Bout Length* | | | | | |
| Day 1 | 10.3 sec | 0.06 | 0.03 | 0.90 | <0.0001 |
| Day 2 | 14.6 sec | **0.006*** | 0.04 | 0.85 | 0.0009 |
| Day 3 | 19.3 sec | **0.0004*** | 0.04 | 0.85 | 0.0001 |
| Day 4 | 19.7 sec | **0.0009*** | 0.04 | **0.16**[#] | **0.57**[#] |
| Day 5 | 21.7 sec | **0.001*** | 0.03 | 0.98 | <0.0001 |
| Day 6 | 14.9 sec | 0.08 | 0.04 | 0.71 | 0.006 |
| Day 7 | 18.3 sec | 0.05 | 0.03 | 0.86 | 0.0003 |
| *Cumulative Sleep* | | | | | |
| Day 1 | 36 min | 0.35 | 0.0005 | – | – |
| Day 2 | 38 min | 0.17 | 0.0006 | – | – |
| Day 3 | 33 min | 0.60 | 0.0004 | – | – |
| Day 4 | 32 min | 0.69 | 0.0004 | – | – |
| Day 5 | 33 min | 0.68 | 0.0004 | – | – |
| Day 6 | 30 min | 0.78 | 0.0004 | – | – |
| Day 7 | 31 min | 0.71 | 0.0004 | – | – |

**Dark Period**

| | **Mean Δ** | **Δ *p*-value** | **Δ *d*** | **ρ** | **ρ *p*-value** |
|---|---|---|---|---|---|
| *Percent Sleep* | | | | | |
| Day 1 | 4.4% | 0.51 | 0.08 | 0.95 | <0.0001 |
| Day 2 | 0.4% | 0.96 | 0.01 | 0.99 | <0.0001 |
| Day 3 | 3.7% | 0.61 | 0.12 | 0.98 | <0.0001 |
| Day 4 | 4.0% | 0.57 | 0.09 | 0.95 | <0.0001 |
| Day 5 | 2.6% | 0.65 | 0.12 | 0.95 | <0.0001 |
| Day 6 | 4.6% | 0.34 | 0.07 | 0.98 | <0.0001 |
| Day 7 | 2.2% | 0.65 | 0.14 | 0.82 | 0.007 |

| | | | | | |
|---|---|---|---|---|---|
| *Mean Bout Length* | | | | | |
| Day 1 | 4.8 sec | 0.42 | 0.07 | 0.96 | <0.0001 |
| Day 2 | 2.0 sec | 0.76 | 0.05 | 0.96 | <0.0001 |
| Day 3 | 1.8 sec | 0.79 | 0.03 | 0.89 | 0.001 |
| Day 4 | 0.4 sec | 0.95 | 0.02 | 0.96 | <0.0001 |
| Day 5 | 1.1 sec | 0.87 | 0.05 | 0.87 | 0.003 |
| Day 6 | 0.4 sec | 0.94 | 0.005 | 0.92 | 0.0005 |
| Day 7 | 0.1 sec | 0.98 | 0.06 | 0.78 | 0.01 |
| *Cumulative Sleep* | | | | | |
| Day 1 | 31 min | 0.99 | 0.004 | – | – |
| Day 2 | 5 min | 0.99 | 0.001 | – | – |
| Day 3 | 8 min | 0.99 | 0.002 | – | – |
| Day 4 | 15 min | 0.99 | 0.004 | – | – |
| Day 5 | 13 min | 0.99 | 0.004 | – | – |
| Day 6 | 12 min | 0.99 | 0.005 | – | – |
| Day 7 | 13 min | 0.99 | 0.004 | – | – |

**Table S5:** Differences between estimated marginal means (Δ), corresponding *p*-values, and effect sizes (*d*) from generalized linear mixed models for percent sleep, mean bout length, and cumulative sleep during days 1, 2, 3, and 4 following the first LPS administration (LPS1) compared to sleep following the second LPS administration (LPS2). Comparisons are made within treatment group (e.g., control LPS1 vs. control LPS2). Estimated nonparametric Spearman's rank correlation (ρ) and corresponding *p*-values for temporal pattern differences in percent sleep and mean bout length. * indicates statistically significant differences in means; # indicates statistically significant temporal pattern differences.

**Light Period**

| | **Mean Δ** | **Δ *p*-value** | **Δ *d*** | **ρ** | **ρ *p*-value** |
|---|---|---|---|---|---|
| *Percent Sleep* | | | | | |
| Day 1 – Control | 0.4% | 0.95 | 0.02 | 0.67 | 0.01 |
| Day 1 – PLX | 3.6% | 0.48 | 0.05 | **0.39#** | **0.17#** |
| Day 2 – Control | 0.9% | 0.84 | 0.02 | **0.52#** | **0.06#** |
| Day 2 – PLX | 2.2% | 0.68 | 0.003 | 0.73 | 0.004 |
| Day 3 – Control | 0.0% | 0.99 | 0.07 | **0.01#** | **0.98#** |
| Day 3 – PLX | 1.1% | 0.78 | 0.006 | 0.67 | 0.01 |
| Day 4 – Control | 2.2% | 0.60 | 0.04 | **0.28#** | **0.33#** |
| Day 4 – PLX | 3.4% | 0.42 | 0.01 | **-0.05#** | **0.87#** |
| *Mean Bout Length* | | | | | |
| Day 1 – Control | 19.7 sec | **<0.0001*** | 0.07 | **-0.16#** | **0.58#** |
| Day 1 – PLX | 30.2 sec | **<0.0001*** | 0.13 | **-0.35#** | **0.23#** |
| Day 2 – Control | 6.4 sec | **0.03*** | 0.05 | **0.34#** | **0.22#** |
| Day 2 – PLX | 17.4 sec | **0.0006*** | 0.10 | **0.59#** | **0.07#** |
| Day 3 – Control | 0.2 sec | 0.97 | 0.01 | **0.24#** | **0.42#** |
| Day 3 – PLX | 26.2 sec | **<0.0001*** | 0.10 | 0.64 | 0.02 |
| Day 4 – Control | 1.7 sec | 0.68 | 0.01 | **0.22#** | **0.44#** |
| Day 4 – PLX | 19.5 sec | **0.002*** | 0.10 | 0.64 | 0.02 |
| *Cumulative Sleep* | | | | | |
| Day 1 – Control | 1 min | 0.99 | 0.0001 | – | – |
| Day 1 – PLX | 30 min | 0.95 | 0.0001 | – | – |
| Day 2 – Control | 8 min | 0.99 | 0.0001 | – | – |
| Day 2 – PLX | 28 min | 0.97 | 0.0001 | – | – |
| Day 3 – Control | 2 min | 0.99 | 0.0001 | – | – |
| Day 3 – PLX | 21 min | 0.99 | 0.0001 | – | – |
| Day 4 – Control | 17 min | 0.99 | 0.0001 | – | – |
| Day 4 – PLX | 36 min | 0.89 | 0.0001 | – | – |

**Dark Period**

| | **Mean Δ** | **Δ *p*-value** | **Δ *d*** | **ρ** | **ρ *p*-value** |
|---|---|---|---|---|---|
| *Percent Sleep* | | | | | |
| Day 1 – Control | 7.6% | 0.18 | 0.09 | **0.60#** | **0.06#** |
| Day 1 – PLX | 17.5% | **0.004*** | 0.07 | 0.89 | 0.001 |

| | | | | | |
|---|---|---|---|---|---|
| Day 2 – Control | 5.9% | 0.49 | 0.10 | 0.78 | 0.01 |
| Day 2 – PLX | 16.9% | **0.01*** | 0.04 | 0.81 | 0.008 |
| Day 3 – Control | 5.2% | 0.54 | 0.04 | 0.81 | 0.008 |
| Day 3 – PLX | 22.3% | **0.004*** | 0.24 | 0.83 | 0.006 |
| Day 4 – Control | 2.3% | 0.75 | 0.10 | 0.94 | <0.0001 |
| Day 4 – PLX | 16.1% | **0.01*** | 0.19 | 0.94 | <0.0001 |
| *Mean Bout Length* | | | | | |
| Day 1 – Control | 11.9 sec | **0.007*** | 0.05 | 0.78 | 0.01 |
| Day 1 – PLX | 5.0 sec | 0.35 | 0.04 | **0.53#** | **0.12#** |
| Day 2 – Control | 5.4 sec | 0.40 | 0.04 | 0.88 | 0.002 |
| Day 2 – PLX | 3.9 sec | 0.45 | 0.02 | 0.79 | 0.01 |
| Day 3 – Control | 6.8 sec | 0.35 | 0.03 | 0.90 | 0.0008 |
| Day 3 – PLX | 14.2 sec | **0.04*** | 0.06 | 0.88 | 0.002 |
| Day 4 – Control | 0.0 sec | 0.99 | 0.03 | 0.89 | 0.001 |
| Day 4 – PLX | 12.8 sec | 0.05 | 0.06 | 0.95 | <0.0001 |
| *Cumulative Sleep* | | | | | |
| Day 1 – Control | 52 min | 0.11 | 0.004 | – | – |
| Day 1 – PLX | 116 min | **<0.0001*** | 0.01 | – | – |
| Day 2 – Control | 37 min | 0.38 | 0.003 | – | – |
| Day 2 – PLX | 107 min | **<0.0001*** | 0.01 | – | – |
| Day 3 – Control | 30 min | 0.23 | 0.004 | – | – |
| Day 3 – PLX | 130 min | **<0.0001*** | 0.01 | – | – |
| Day 4 – Control | 13 min | 0.99 | 0.001 | – | – |
| Day 4 – PLX | 109 min | **<0.0001*** | 0.01 | – | – |

**Table S6:** Differences between estimated marginal means (Δ), corresponding *p*-values, and effect sizes (*d*) from generalized linear mixed models for number of microglia branches, microglia branch length, and endpoints per branch. * indicates statistically significant differences in means.

| Microglia Skeletal Analysis | | | |
|---|---|---|---|
| | **Mean Δ** | **Δ *p*-value** | **Δ *d*** |
| *Microglia Cells* | | | |
| Treatment | 0 | 0.60 | 0.01 |
| *Number of Processes* | | | |
| Treatment | 13 | 0.08 | 0.005 |
| *Branch Length* | | | |
| Treatment | 28 µm | 0.40 | 0.002 |
| *Endpoints/Microglia* | | | |
| Treatment | 18 | **0.002*** | 0.01 |
